# Iota-Carrageenan is a potent inhibitor of rhinovirus infection

**DOI:** 10.1186/1743-422X-5-107

**Published:** 2008-09-26

**Authors:** Andreas Grassauer, Regina Weinmuellner, Christiane Meier, Alexander Pretsch, Eva Prieschl-Grassauer, Hermann Unger

**Affiliations:** 1 Marinomed Biotechnologie GmbH, Veterinaerplatz 1/HA, A-1210 Vienna, Austria; 2 Laboratory of Tropical Veterinary Medicine, Veterinary University Vienna, Veterinaerplatz 1, A-1210 Vienna, Austria

## Abstract

**Background:**

Human rhinoviruses (HRVs) are the predominant cause of common cold. In addition, HRVs are implicated in the worsening of COPD and asthma, as well as the loss of lung transplants. Despite significant efforts, no anti-viral agent is approved for the prevention or treatment of HRV-infection.

**Results:**

In this study we demonstrate that Iota-Carrageenan, a sulphated polysaccharide derived from red seaweed, is a potent anti-rhinoviral substance in-vitro. Iota-Carrageenan reduces HRV growth and inhibits the virus induced cythopathic effect of infected HeLa cells. In addition, Iota-Carrageenan effectively prevents the replication of HRV1A, HRV2, HRV8, HRV14, HRV16, HRV83 and HRV84 in primary human nasal epithelial cells in culture. The data suggest that Iota-Carrageenan acts primarily by preventing the binding or the entry of virions into the cells.

**Conclusion:**

Since HRV infections predominately occur in the nasal cavity and the upper respiratory tract, a targeted treatment with a product containing Iota-Carrageenan is conceivable. Clinical trials are needed to determine whether Iota-Carrageenan-based products are effective in the treatment or prophylaxis of HRV infections.

## Background

The family Picornaviridae comprises some notable members, including human rhinovirus (HRV), which infects humans more frequently than any other virus. Infections with HRV lead to the common cold with symptoms such as sore throat, rhinitis, nasal congestion, and cough [1]. The National Institutes of Health (NIH) estimates that there are more than a billion cases of common colds in the USA each year. Besides the self-limiting infection, HRV is implicated as a cause or predisposing agent for otitis media, sinusitis and exacerbations of asthma, as well as other lower respiratory tract disorders [1-4].

Despite significant efforts no anti-viral agent is approved for the prevention or treatment of HRV-infection. A number of anti-viral compounds have been evaluated for the management of HRV induced colds, including the capsid binders pirodavir and Pleconaril [3,5-7]. Studies with biologicals such as intranasal Tremacamra a soluble intercellular adhesion molecule 1 (ICAM-1) and alpha interferon have shown that targeting HRV is possible especially when the drugs are applied prophylactically or the intervention is early. [8-10].

Another approach targets the HRV proteases 2A and 3C with small molecules. Protease 3C is an enzyme necessary for the posttranslational cleavage of viral precursor polyproteins. Studies with experimental HRV infection showed promising results for Ruprintrivir a compound developed by (Agouron/Pfizer) [6]. Development of Tremacamra and Ruprintrivir has not advanced to phase III clinical trials until today.

To effectively inhibit the HRV induced inflammatory cascade of the common cold the treatment needs to be initiated rapidly after the first symptoms or even before. Since the HRV infection is self limiting and not life threatening in most cases a potential therapy has to be safe and effective with an almost unrecognizable level of side effects.

Polymers from various sources are substances that might bear these desired safety properties. In particular sulphated polysaccharides including Carrageenan, a sulphated polysaccharide extracted from red seaweed has an excellent safety profile and has shown anti-viral efficacy against several viruses. The anti-HIV-1 activity of Lambda-, Kappa- and Iota-Carrageenan and other sulphated polymers has been described previously [11,12]. In a review, Gonzalez M.E. et al. [11] report an anti-viral efficacy of different sulphated polysaccharides including Iota-Carrageenan against several animal viruses. Iota-Carrageenan showed anti-viral activity against the enveloped viruses Herpes simplex virus type 1 and type 2, Semliki Forest virus (SFV), vaccinia virus, African swine fever virus (ASF), and against encephalomyocarditis (EMC) virus. Iota-Carrageenan had no effect on vesicular stomatitis virus (VSV), measles virus, polio virus type 1 (member of the picornaviridae) and adenovirus type 5. Carlucci et al. [11,13] demonstrated a protective effect of Lambda-Carrageenan on genital herpes simplex virus infection in mice. Pujol et al. [14] showed the anti-viral activity of a Carrageenan isolated from Gigartina skottsbergii against intraperitoneal murine herpes simplex virus infection.

Carrageenan has been generally recognized as safe by the FDA. In addition, Carrageenan has been extensively used in the food, cosmetic and pharmaceutical industry as a thickener and gelling agent. In this report we show that Iota-Carrageenan inhibits the replication of HRV in tissue culture. Therefore Iota-Carrageenan might be a promising candidate for the evaluation of efficacy against HRV in clinical trials in humans.

## Results

### Carrageenan promotes cell survival after HRV2 infection

**Figure 1:**
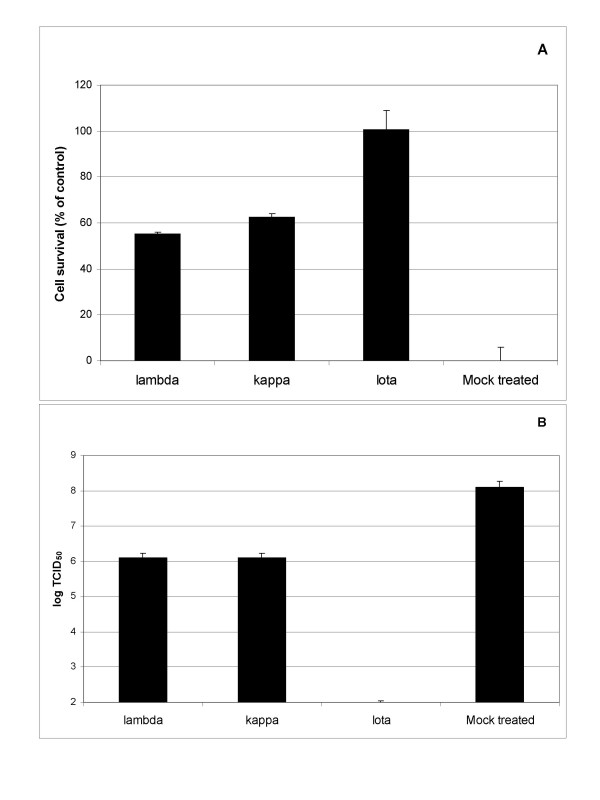
**Carrageenans promote cell viability of HRV2 infected HeLa cells and inhibit HRV2 replication in vitro**. A. HeLa cells grown in 96-well plates were infected with HRV2 (0,1 TCID_50_/cell) in the presence of Carrageenans (different types are indicated at the x-axis) at a concentration of 200 μg/ml. Plates were incubated at 37°C until cells in the control (no polymer added) showed >90% damage. Cell proliferation was determined with an XTT-assay. OD values (492 nm) obtained from mock infected cells (compare x-axis) were set to 100%, and the viability of cells infected in the absence of polymer was set to 0% (y-axis). The bars represent the mean of a quadruplicate experiment, the standard deviation is indicated. B. HeLa cells in 24-well plates were infected with HRV2 (0,1 TCID_50_/cell) in the presence of Carrageenans (different types are indicated at the x-axis) at a concentration of 200 μg/ml. Viral infectivity in the supernatants was determined by TCID_50 _assay on HeLa cells (y-axis). Values represent the mean of six parallel titrations, standard deviation is indicated.

Carrageenan has been shown to bear anti-viral activity against herpes simplex virus (HSV), cytomegalovirus (CMV), dengue virus, papilloma virus, and human immunodeficiency virus (HIV) [11,12,15,16]. To study the effect of different Carrageenan-subtypes (Lambda-, Kappa-, and Iota-Carrageenan) on HRV, a comparative experiment was performed. HRV infection of cells induces morphological changes and cell death, commonly known as cythopathic effect (CPE). To quantify the virus induced cell death, a proliferation assay was employed. As indicator for cell survival the tetrazolium substrate conversion into a formazan dye was measured (XTT-Test). The resulting optical density (OD) values reflected the metabolic activity of cells. HeLa cells were infected with HRV2 at an amount of input virus of 0,1 TCID_50_/cell. Metabolic activity was measured 48 hours post infection (p.i.), when a CPE of more than 90% was observed by microscopy. OD values of HRV2 infected, untreated HeLa cells were set to 0%. Survival of mock infected cells was set to 100%. Polymers Lambda-, Kappa-, and Iota-Carrageenan were applied at a concentration of 200 μg/ml. The protection against virus-induced cell death was 55% for Lambda-Carrageenan and 62% for Kappa-Carrageenan (Figure. 1A). However, 200 μg/ml Iota-Carrageenan completely blocked the virus induced cell death. All tested Carrageenans did not show cytotoxic effects on uninfected cells up to concentrations of 1000 μg/ml after 48 h (data not shown).

### Iota-Carrageenan reduces production of HRV particles

HeLa cells were seeded in 24-well plates (2 * 10^4 ^cells per well) and infected with HRV2 (0,1 TCID_50_/cell) in the presence of Iota-Carrageenan at a concentration of 200 μg/ml. When cell lysis was observed in the untreated control, supernatants were harvested. Viral titers were determined by TCID_50 _assays on HeLa cells. HRV2 replication in untreated control cells resulted in the generation of 10^8 ^TCID_50_/ml after 48 h (Figure 1B). Lambda- and Kappa-Carrageenan reduced HRV2 titers in cell supernatants by two orders of magnitude. Iota-Carrageenan exceeded the activity of Lambda- and Kappa-Carrageenan and prevented viral titer production for at least 6 orders of magnitude when compared with the untreated control (Figure 1B). Since, the detection limit was 10^2 ^TCID_50 _in this test an even higher effect cannot be excluded.

### The anti-viral effect of Iota-Carrageenan is dependent on the amount of input virus

**Figure 2:**
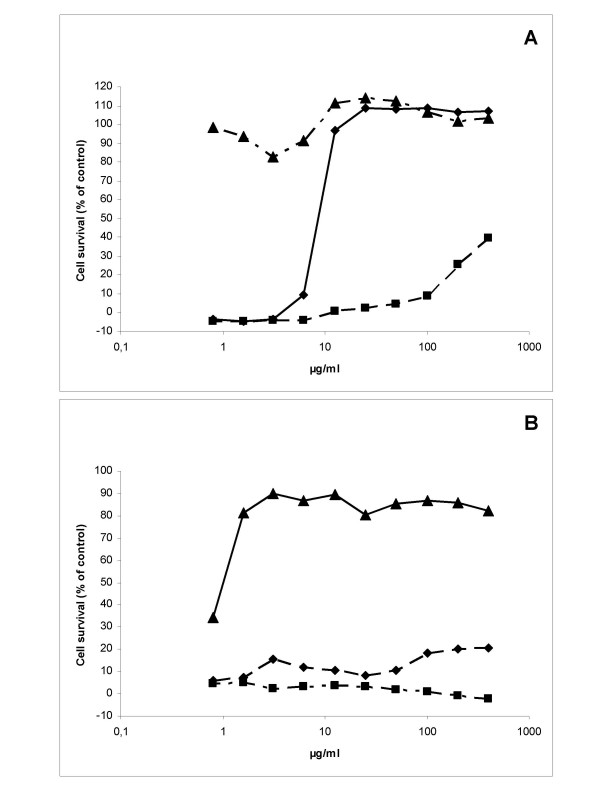
**Iota-Carrageenan induced inhibition of HRV2 infected cells is dependent on the amount of virus**. A. Preincubation of virus with polymer. HeLa cells grown in 96-well plates were infected with HRV2 in the presence of Iota-Carrageenan at concentrations as indicated at the x-axis. B. Treatment with polymer after infection. HeLa cells grown in 96-well plates were infected with HRV2. 30 minutes after infection medium containing Iota-Carrageenan at the concentrations indicated at the x-axis was added. Plates were incubated at 37°C until cells in the control (no polymer added) showed >90% damage. Cell proliferation was determined with an XTT-assay. OD values (492 nm) obtained from mock infected cells (compare x-axis) were set to 100%, and the viability of cells infected in the absence of polymer was set to 0% (y-axis). Black triangles indicate an amount of input virus of 0,01 TCID_50_/cell, black diamonds indicate 0,1 TCID_50_/cell and black squares indicate 1 TCID_50_/cell. A representative experiment is shown.

To test whether the amount of input virus has an effect on the anti-viral properties of Iota-Carrageenan, HeLa cells were infected with HRV2 with three different amounts of input virus. The survival of infected HeLa cells after 72 h was determined with an XTT assay as described above. In one arm of the experiment the infection was performed in the presence of Iota-Carrageenan (prophyalxis experiment). In the other arm of the experiment the infection was performed in the absence of polymer (treatment experiment). 30 minutes after infection the inocula were removed in both groups, cells were washed and polymer was added at concentrations ranging from 0,7 μg/ml to 400 μg/ml. A clear dependency on the amount of input virus was observed in both tested schemes (Figure 2). At an amount of input virus of 0,01 TCID_50_/cell HeLa cells were completely protected from virus induced CPE at a concentration as low as 4 μg/ml in both the prophylaxis and the treatment experiment. At 0,1 TCID_50_/cell the protective concentration of Iota-Carrageenan increased to 10 μg/ml in the prophylaxis experiment and no protection was observed in the treatment experiment. With input virus of 1 TCID_50_/cell in the prophylaxis experiment only the highest concentration of Iota-Carrageenan (400 μg/ml) resulted in a partial protection and again no effect was observed in the treatment experiment. These data suggest that the anti-viral effect of Iota-Carrageenan is dependent on the amount of input virus. In addition, the polymer is more efficacious in a prophylactic setting compared to a treatment setting.

**Figure 3:**
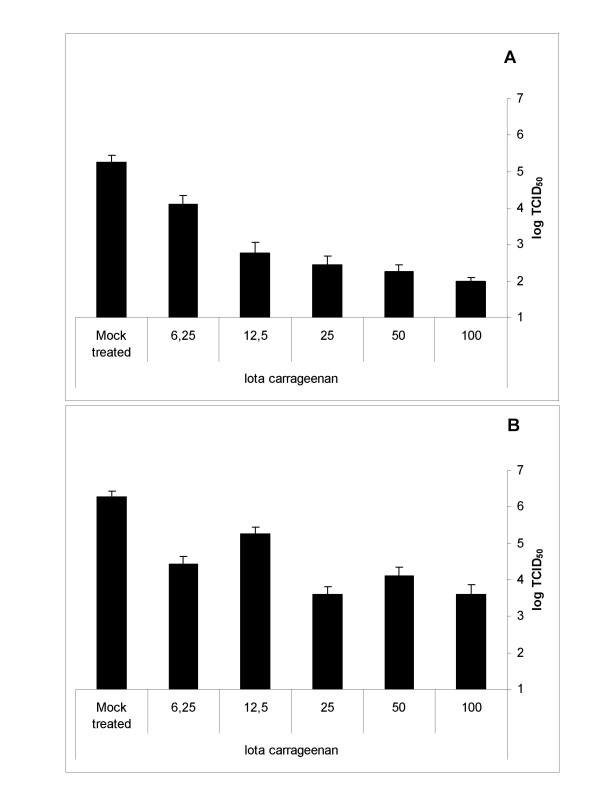
**Iota-Carrageenan dose-dependently inhibits HRV2 replication in cell culture**. (A) Preincubation of virus with polymer. HeLa cells grown in 12-well plates were infected with HRV2 (0,1 TCID_50_/cell) in the presence of Iota-Carrageenan at the concentration indicated at the x-axis. 30 minutes after infection the inoculum was removed and medium containing Iota-Carrageenan with the concentration indicated was added. Untreated cells were used as control (mock treated). B. Treatment with polymer after infection. HeLa cells grown in 24-well plates were infected with HRV2 (0,1 TCID_50_/cell). 30 minutes after infection the inoculum was removed and medium containing Iota-Carrageenan with the concentration indicated at the x-axis was added. Untreated cells were used as control (mock treated). Viral titers in the supernatants of infected cells were determined after 48 h by TCID_50 _assay on HeLa cells. Values are the means from six parallel titrations, standard deviation is indicated.

In an analogous experiment the viral titers in supernatant were determined. Cells were infected with HRV2 (0,1 TCID_50_/cell) in the presence or absence of different concentrations of Iota-Carrageenan. 48 h after infection supernatants were harvested and titers were determined with a TCID_50 _assay. When the infection was done in presence of Iota-Carrageenan, a dose-dependent reduction of the viral titer was observed showing a reduction of 3 orders of magnitude at 100 μg/ml (Figure 3A). When the polymer was added after the infection a reduction in the viral titer of 2 orders of magnitude was observed for the concentrations 25, 50 and 100 μg/ml. At the concentrations 12,5 and 6,25 μg/ml the reduction in the viral titer was less pronounced (Figure 3B). Similar to the CPE assays, this experiment showed that titers of supernatants of cells infected in the presence of Iota-Carrageenan are reduced compared to titers of supernatants of cells infected in the absence of Iota-Carrageenan. In order to exclude a direct effect of the polymer to the cells we incubated Hela cells with Iota-Carrageenan three hours before infection. Cells were washed twice with PBS prior to infection with HRV. This treatment of the cells did not result in a significant effect on cell survival and replication of HRV (data not shown). The data imply that the anti-viral effect of Iota-Carrageenan against HRV is due to the inhibition of viral binding or entry into the cells.

### Search for Iota-Carrageenan resistant variants

**Figure 4:**
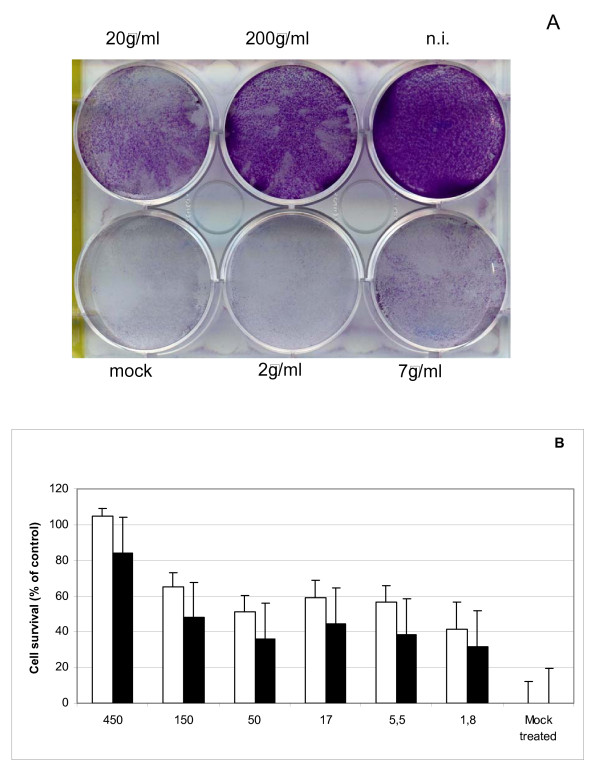
**Iota-Carrageenan does not induce HRV2 escape mutants after 10 passages**. A. HeLa cells in 6-well plates (8 * 10^4 ^cells per well) were infected with HRV2 in the presence of Iota-Carrageenan. After infection the cells were washed and medium containing polymer was added at concentrations between 2 μg/ml and 100 μg/ml. Plates were incubated at 37°C until cells in the control (no polymer added) showed >90% damage. Living cells were fixed and stained with crystal violet staining solution. B. Supernatants from infected wells with Carrageenan of 20 μg/ml were used for the next infection round. For the following infection rounds the supernatants of wells with 7 μg/ml or 20 μg/ml were used for the subsequent infection round. After ten repetitive infection experiments the sensitivity of the resulting virus (white bars) to different concentrations of Iota-Carrageenan (x-axis) was compared with that of the original virus (black bars). Cell proliferation was determined with an XTT-assay. Survival of mock infected cells was set to 100%, and that in the absence of polymer was set to 0% (y-axis). The bars represent means of six independent experiments standard deviation is indicated.

We were interested whether Iota-Carrageenan resistant variants occur with high frequency and can be characterized. 6-well plates with HeLa cells (8 * 10^4 ^cells per well) were infected in the presence of polymer with HRV2 (0,1 TCID_50_/cell) and 30 minutes post infection medium containing a given polymer concentration was added. After two days of incubation at 37°C a cytopathic effect was observed in the mock treated control and supernatants were collected. The supernatants of those wells showing partial protection from virus induced cells death (7 μg/ml and 20 μg/ml) were used as inoculum for the next selection round. After ten repetitive infection experiments, the original HRV2 virus was compared with the HRV2 virus from the last passage in a CPE inhibition experiment (for details see material and methods; Figure 4). No significant difference was observed in a CPE inhibition experiment between the original HRV2 viruses that have been obtained after ten passages. This result indicates that escape mutants against Iota-Carrageenan do not occur frequently in HeLa cells.

### Iota-carageenan blocks replication of HRV2 in primary human nasal epithelial cells (HNep)

**Figure 5:**
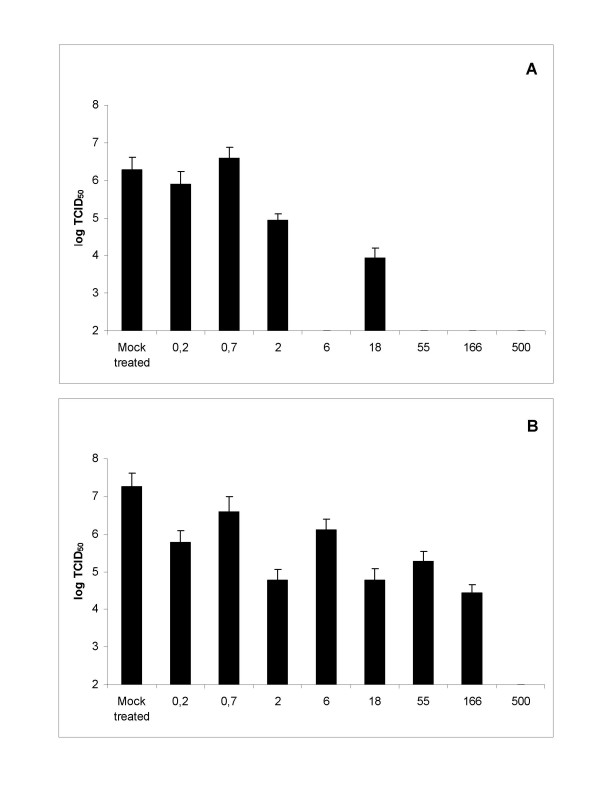
**Effect of Iota-carageenan on HRV2 infected human nasal epithelial cells**. A. Preincubation of virus with polymer. HNep cells were grown in 24-well plates were infected with HRV2 (0,1 TCID_50_/cell) in the presence of Iota-Carrageenan at the concentration indicated at the x-axis. 30 minutes after infection the inoculum was removed and medium containing Iota-Carrageenan with the concentration indicated was added. B. Treatment with polymer after infection. HNep cells were grown in 24-well plates were infected with HRV2 (0,1 TCID_50_/cell). 30 minutes after infection the inoculum was removed and medium containing Iota-Carrageenan with the concentration indicated at the x-axis was added. Viral titers in the supernatants of infected cells were determined after 48 h by TCID_50 _assay on HeLa cells (y-axis). Bars represent means of four parallel experiments, standard deviation is indicated.

In order to study whether the activity against HRV is a tissue culture phenomenon an experiment with human nasal epithelial cells (HNep) was conducted. HNep cells were grown in 24-well plates. The infection with HRV2 (0,1 TCID_50_/cell) was carried out in the presence or absence of Iota-Carrageenan and 30 minutes post infection medium containing polymer in the range of 0,2 μg/ml to 500 μg/ml was added. After 48 hours analysis of viral titers in the supernatants of infected cells revealed that HRV2 replicates to titers of approximately 10^7 ^TCID_50_/ml on HNep cells. The viral titer was below the detection limit of 10^2 ^TCID_50_/ml when 55 μg/ml of Iota-Carrageenan was already present during the infection (Figure 5A). When the infection was done in the absence of Iota-Carrageenan a concentration of 500 μg/ml was needed to reduce the viral replication below the detection limit (Figure 5B). However, in both cases a significant reduction in the viral titer was observed when the polymer concentration was at least 2 μg/ml. This result shows that Iota-Carrageenan inhibits replication of HRV2 on HNep cells.

### Iota-carrageenan inhibits replication of HRV serotypes 1A, 8, 14, 16, 83, 84 on primary human epithelial cells

**Figure 6:**
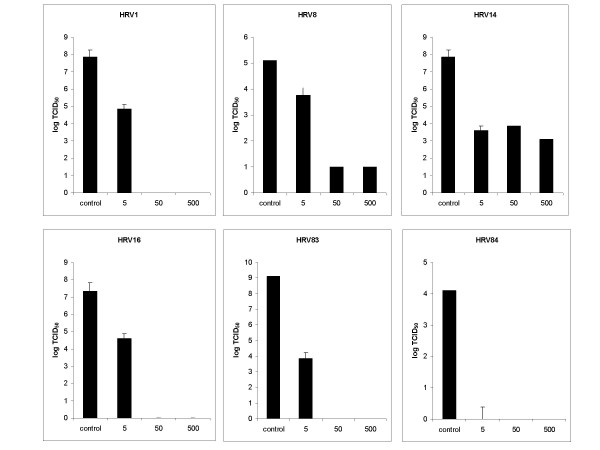
**Effect of Iota carageenan on the replication of HRVstrains 1A, 2, 8, 14, 16, 39, 83 and 84 on human nasal epithelial cells**. HNep cells were grown in 96-well plates were infected with different HRV strains (indicated at the top of each panel; 0,1 TCID_50_/cell) in the presence of Iota-Carrageenan at the concentrations indicated at the x-axis. 30 minutes after infection the inoculum was removed and medium containing Iota-Carrageenan with the same concentration was added. Viral titers in the supernatants of infected cells were determined after 48 h by TCID_50 _assay on HeLa cells (y-axis). Bars represent means from four parallel experiments, standard deviations are indicated.

Since more than 100 distinctive HRV serotypes are circulating in humans it was important to reveal whether Iota-Carrageenan is also effective against other strains of HRVs. The work of Ledford et al. shows that the EC_50 _concentration against HRV of the capsid binder Pleconaril has a strain dependent variability between 0,01 μg/ml and >12,5 μg/ml [17]. Based on this work we selected HRV1A, HRV16 and HRV8 for testing. These three viruses belong to the HRV-A virus group and are in contrast to HRV2 relatively insensitive to Pleconaril. From the HRV-B virus group we tested the Pleconaril sensitive strain HRV83, the moderate sensitive strain HRV14, and HRV84, a strain that cannot be inhibited by Pleconaril at a concentration of 12,5 μg/ml. Primary human nasal epithelial cells were seeded in 96-well plates (4.8 * 10^3 ^cells per well) and infected with HRV an amount of input virus of 2 TCID_50_/cell. Supernatants were harvested between 48–72 hours after infection and the viral titers were determined by TCID_50 _assays on HeLa cells. While HRV1, HRV14, HRV16 and HRV83 replicated to titers above 10^7 ^TCID_50_/ml, HRV8 reached a titer of 10^5,1 ^TCID_50_/ml and HRV84 a titer of 10^4,1 ^TCID_50_/ml (Figure 6). A Iota-Carrageenan concentration of 50 μg/ml was sufficient to reduce the replication on HNep cells of all tested viruses by more than 3 orders of magnitude (99,9%). At a Iota-Carrageenan concentration of 5 μg/ml an inhibition of greater than 99% was observed for HRV1, HRV14, HRV16, HRV83 and HRV84. However, for HRV8 a reduction from 10^5,2 ^TCID_50_/ml to 10^3,8 ^TCID_50_/ml was observed at 5 g/ml Iota-Carrageenan. No toxic effects have been observed on HNep cells at the highest tested Iota-Carrageenan concentration of 500 μg/ml. This result demonstrates that Iota-Carrageenan can effectively block the replication of six distinct HRV strain on HNEp cells.

## Discussion

In this report we demonstrate that Iota-Carrageenan, a commercial thickening agent derived from seaweed, is a potent inhibitor of rhinovirus infectivity in vitro. Two other related polymers Lambda- and Kappa-Carrageenan show moderate effects and did not fully inhibit virus induced cell death in HRV2 infected HeLa cells (Figure 1).

Protection of HeLa cells from virus induced cell death was dependent on the amount of input virus in both cases, when cells were infected in the presence or absence of Iota-Carrageenan (Figure 2). When cells were treated after infection, protection was observed only for the lowest input amount tested (0,01 TCID_50_/cell; Figure 2B). Therefore we conclude that Iota-Carrageenan most likely inhibits binding or entry of the virus into the cells and not a later stage of viral replication. These findings are consistent with previous studies with other viruses that have shown that Carrageenan is active against several viruses in vitro and in vivo [11,16,18-24].

Iota-Carrageenan has been shown to be a potent inhibitor of papillomavirus infection with 50% inhibitory doses in the low ng/ml range [12]. However, when tested against rhinoviruses Iota-Carrageeenan appears to be effective against HRV at concentrations several orders of magnitude higher in the low μg/ml range (Figure 3). This result is comparable with in-vitro data of other viruses such as HIV-1 and Herpes virus [15,25].

Repeated passage of the HIV virus in the presence of polyanions can lead to resistance mediated by mutations in the envelope glycoprotein gp120, particularly in the V3 loop (K269E, Q278H, N293D), as originally shown for dextran sulphate, and subsequently for Zintevir and negatively charged albumins [26,27]. While resistant variants emerge relatively fast with HIV-1 we were not able to detect a difference in an in-vitro test between the original virus stock and a HRV2 virus after 10 subsequent passages in the presence of Iota-Carrageenan at concentrations between 7 μg/ml and 20 μg/ml (Figure 4). Although the potential emergence of resistant variants deserves detailed and extensive studies we conclude that Iota-Carrageenan resistant variants do not occur with a high frequency. This result supports the hypothesis that Iota-Carrageenan prevents HRV virions from cell attachment or cell entry in a less specific manner when compared to the results that were obtained by Buck and co-workers for papillomavirus [12]. However, it cannot be excluded that resistant variants of HRV2 may occur at later passages and further studies are needed.

In situ hybridization studies have revealed that the airway epithelial cell is the primary site of HRV infection in vivo [28,29] and there is growing evidence that virally induced alterations in epithelial cell biology may contribute to disease pathogenesis [30,31]. Thus we selected HNep cells as target cells for rhinovirus infection studies. Again Iota-Carrageenan was found to be effective against HRV2 on primary human epithelial cells with similar results when compared to the studies on HeLa cells (Figure 5). Our study also shows that viral titers in supernatants of infected HNep cells can vary by several orders of magnitude dependent on the strain (Figure 6). Replication studies with three Type A viruses HRV1A, HRV8, HRV16 and three Type B viruses HRV14, HRV83 and HRV84 revealed that Iota-Carrageenan is effectively inhibiting replication of Type A and Type B rhinoviruses when the polymer is present during infection (Figure 6). Differences between batches of HNep cells resulted in a variation of titers of HRV strains tested. However the anti-viral activity of Iota-Carrageenan was comparable in all tested HNep batches (data not shown).

Our data on primary cells are consistent with the data from infected HeLa cells and thereby support the hypothesis that Iota-Carrageenan interferes with viral replication at a very early stage of viral infection. Most likely, the binding of virions to the cells is hindered. It is not clear whether Carrageenan exerts any additional effects. The inhibitory effect of Iota-Carrageenan might be due to the occlusion of virion surfaces involved in binding to cellular receptors. Alternatively, obligatory conformational changes in the virus may be blocked and in addition, a post-attchment inhibitory effect may exist as described recently for papillomaviruses [12,32].

Rhinovirus infections yearly account for huge economic losses in terms of lost school and working days. Moreover, recent evidence points towards rhinoviruses as a major cause in exacerbating asthma and COPD. For a review see Drescher et al 2007 [33]. A number of molecules and strategies have been examined in order to combat rhinovirus and the whole family of picornaviruses [5,12]. Despite this, no therapy has been approved for the treatment of rhinovirus infections yet, and patient care remains symptomatic.

Moreover, the efforts in therapeutic development are hampered by the fact that more than 100 distinctive HRV serotypes are circulating in humans. Our studies on primary HNep cells demonstrate that Iota-Carrageenan potently inhibits the replication of the seven distinct rhinovirus strains HRV1, 2, 8, 14, 16, 83 and 84 (Figure 5 and Figure 6). Although we are convinced that the result can be extrapolated for the whole family of human rhinoviruses further experiments are needed proof the efficacy on all strains of HRV.

## Conclusion

The primary site of infection and replication of HRV in humans is the nasal mucosa. It is tempting to speculate that a targeted treatment of the nasal mucosa with Iota-Carrageenan might create a hostile environment for HRV and thereby block viral entry and replication. Carrageenan is generally recognized as safe for use in food and topical applications. Given the sensitivity and anti-viral effectiveness against several strains of HRV in primary human epithelial cells Iota-Carrageenan deserves consideration as a candidate for clinical trials for the prophylaxis and treatment of rhinovirus induced common cold.

## Methods

### Polymers

Lambda carrageen, Kappa carrageen and Iota carrageen were purchased from FMC Biopolymers (Philadelphia, PA). The dry polymer powders were dissolved in cell culture water (PAA, Austria) to a final concentration of 0.4%. This stock solution was sterile filtered through a 0.45 μm filter (Sarstedt, Germany) and stored at 4°C until use.

### Viruses, cell lines and media

HRV serotypes (HRV 1A, 2, 8, 14, 16, 83, 84) were obtained from the American Type Culture Collection (Manassas, VA) and grown on HeLa cells. The stocks were frozen at -80°C and virus titers were determined by TCID_50 _assay. The human cervical epithelial carcinoma cell line (HeLa) was obtained from the American Type Culture Collection. The cells were cultivated in Dulbecco's minimal essential medium (PAA) supplemented with 10% fetal bovine serum (PAA) and 1% antibiotic-antimycotic mix (PAA) in a 37°C incubator (Sanyo, Japan; CO_2_: 5%, relative humidity: >95%). During virus infection and viral experiments a medium containing 2% fetal bovine serum and 1% antibiotic-antimycotic mix was used.

Human nasal epithelial cells were obtained from PromoCell GesmbH (Heidelberg, Germany) and cultivated in airway epithelial cell growth media (PromoCell).

### Inhibition assays

For determination of anti-viral activity a CPE inhibition assay was performed. HeLa cells were seeded in tissue culture plates 24 hours prior the experiments. At 80% confluence cells were infected with inoculums at indicated amounts of input virus (TCID_50_/cell). In order to test whether the polymers can inhibit viral replication the cells were infected with virus in the presence or absence of polymer. For determination of the CPE plates were washed with PBS (PAA) and stained with 1% crystal violet (Sigma, St. Louis, MO) in 20% ethanol (?) and 3.7% formaldehyde (?). Cell damage was quantified with respect to intensity of the stain retained by living cells in a plate reader (Labsystems, Finland) at 630 nm. Alternatively, the cell metabolism was measured with an XTT reagent kit (Roche, Switzerland). Cell survival in the presence of inhibitors was calculated by setting mock infected cells to 100% survival and cells infected without inhibitor to 0% survival. Virus titers in 50% tissue culture infectious doses (TCID_50_)/ml were determined according to Reed and Muench [34].

### Search for resistant variants

HeLa cells (8 * 10^4 ^cells per well) were seeded in a 6-well plate and infected with HRV2. The cells were infected with a polymer virus mixture at final concentrations of 200, 20, 7 and 2 μg/ml with an amount of input virus of 0,1 TCID_50_/cell. As control, one well was mock infected with medium and one well was infected with mock treated virus. After an infection time of 30 minutes the plates were washed twice and an overlay with infection medium containing polymer or control medium was added. Samples were taken from each well when a clear cytopathic effect was visible in the untreated control well. For the next selection round the wells showing a clear CPE and difference to the control infection was used.

The infection cycle was repeated 10 times and the resulting virus sample was compared in a CPE inhibition test with the original virus stock.

## Competing interests

The authors AG, CM, RW, EP, are employed by Marinomed. Authors HU and AP are co-founders of Marinomed. AG, CM, AP, and EP are inventors on patent # WO2008067982 held by Marinomed Biotechnologie GmbH that relates to the content of the manuscript. Marinomed Biotechnologie GmbH is financing the processing charge of this manuscript.

## Authors' contributions

AG, EP and HU participated in design and interpretation of the experiments. RW, CM, AP and AG carried out the viral replication studies on HeLa cells and drafted the manuscript. CM and RW performed the experiments on HNep cells. EP and HU contributed to experimental designs of the study and writing of the manuscript. All authors read and approved of the final manuscript.

## References

[B1] Whitton JL, Cornell CT, Feuer R (2005). Host and virus determinants of picornavirus pathogenesis and tropism. Nat Rev Microbiol.

[B2] Anzueto A, Niederman MS (2003). Diagnosis and treatment of rhinovirus respiratory infections. Chest.

[B3] Brownlee JW, Turner RB (2008). New developments in the epidemiology and clinical spectrum of rhinovirus infections. Curr Opin Pediatr.

[B4] Pitkaranta A, Hayden FG (1998). Rhinoviruses: important respiratory pathogens. Ann Med.

[B5] De Palma AM, Vliegen I, De CE, Neyts J (2008). Selective inhibitors of picornavirus replication. Med Res Rev.

[B6] Hayden FG, Turner RB, Gwaltney JM, Chi-Burris K, Gersten M, Hsyu P, Patick AK, Smith GJ, Zalman LS (2003). Phase II, randomized, double-blind, placebo-controlled studies of ruprintrivir nasal spray 2-percent suspension for prevention and treatment of experimentally induced rhinovirus colds in healthy volunteers. Antimicrob Agents Chemother.

[B7] Hayden FG, Herrington DT, Coats TL, Kim K, Cooper EC, Villano SA, Liu S, Hudson S, Pevear DC, Collett M, McKinlay M (2003). Efficacy and safety of oral pleconaril for treatment of colds due to picornaviruses in adults: results of 2 double-blind, randomized, placebo-controlled trials. Clin Infect Dis.

[B8] Gwaltney JM, Winther B, Patrie JT, Hendley JO (2002). Combined antiviral-antimediator treatment for the common cold. J Infect Dis.

[B9] Hayden FG, Gwaltney JM (1984). Intranasal interferon-alpha 2 treatment of experimental rhinoviral colds. J Infect Dis.

[B10] Hayden FG, Albrecht JK, Kaiser DL, Gwaltney JM (1986). Prevention of natural colds by contact prophylaxis with intranasal alpha 2-interferon. N Engl J Med.

[B11] Gonzalez ME, Alarcon B, Carrasco L (1987). Polysaccharides as antiviral agents: antiviral activity of carrageenan. Antimicrob Agents Chemother.

[B12] Buck CB, Thompson CD, Roberts JN, Muller M, Lowy DR, Schiller JT (2006). Carrageenan is a potent inhibitor of papillomavirus infection. PLoS Pathog.

[B13] Carlucci MJ, Scolaro LA, Noseda MD, Cerezo AS, Damonte EB (2004). Protective effect of a natural carrageenan on genital herpes simplex virus infection in mice. Antiviral Res.

[B14] Pujol CA, Scolaro LA, Ciancia M, Matulewicz MC, Cerezo AS, Damonte EB (2006). Antiviral activity of a carrageenan from Gigartina skottsbergii against intraperitoneal murine herpes simplex virus infection. Planta Med.

[B15] Baba M, Snoeck R, Pauwels R, De CE (1988). Sulfated polysaccharides are potent and selective inhibitors of various enveloped viruses, including herpes simplex virus, cytomegalovirus, vesicular stomatitis virus, and human immunodeficiency virus. Antimicrob Agents Chemother.

[B16] Talarico LB, Pujol CA, Zibetti RG, Faria PC, Noseda MD, Duarte ME, Damonte EB (2005). The antiviral activity of sulfated polysaccharides against dengue virus is dependent on virus serotype and host cell. Antiviral Res.

[B17] Ledford RM, Patel NR, Demenczuk TM, Watanyar A, Herbertz T, Collett MS, Pevear DC (2004). VP1 sequencing of all human rhinovirus serotypes: insights into genus phylogeny and susceptibility to antiviral capsid-binding compounds. J Virol.

[B18] Carlucci MJ, Pujol CA, Ciancia M, Noseda MD, Matulewicz MC, Damonte EB, Cerezo AS (1997). Antiherpetic and anticoagulant properties of carrageenans from the red seaweed Gigartina skottsbergii and their cyclized derivatives: correlation between structure and biological activity. Int J Biol Macromol.

[B19] Carlucci MJ, Scolaro LA, Noseda MD, Cerezo AS, Damonte EB (2004). Protective effect of a natural carrageenan on genital herpes simplex virus infection in mice. Antiviral Res.

[B20] Fernandez-Romero JA, Thorn M, Turville SG, Titchen K, Sudol K, Li J, Miller T, Robbiani M, Maguire RA, Buckheit RW, Hartman TL, Phillips DM (2007). Carrageenan/MIV-150 (PC-815), a combination microbicide. Sex Transm Dis.

[B21] Girond S, Crance JM, Van Cuyck-Gandre H, Renaudet J, Deloince R (1991). Antiviral activity of carrageenan on hepatitis A virus replication in cell culture. Res Virol.

[B22] Hamasuna R, Eizuru Y, Minamishima Y (1994). Inhibition by iota-carrageenan of the spread of murine cytomegalovirus from the peritoneal cavity to the blood plasma. J Gen Virol.

[B23] Neurath AR, Strick N, Li YY (2002). Anti-HIV-1 activity of anionic polymers: a comparative study of candidate microbicides. BMC Infect Dis.

[B24] Talarico LB, Damonte EB (2007). Interference in dengue virus adsorption and uncoating by carrageenans. Virology.

[B25] Witvrouw M, De CE (1997). Sulfated polysaccharides extracted from sea algae as potential antiviral drugs. Gen Pharmacol.

[B26] Cabrera C, Witvrouw M, Gutierrez A, Clotet B, Kuipers ME, Swart PJ, Meijer DK, Desmyter J, De CE, Este JA (1999). Resistance of the human immunodeficiency virus to the inhibitory action of negatively charged albumins on virus binding to CD4. AIDS Res Hum Retroviruses.

[B27] Este JA, Schols D, De VK, Van LK, Vandamme AM, Desmyter J, De CE (1997). Development of resistance of human immunodeficiency virus type 1 to dextran sulfate associated with the emergence of specific mutations in the envelope gp120 glycoprotein. Mol Pharmacol.

[B28] Arruda E, Boyle TR, Winther B, Pevear DC, Gwaltney JM, Hayden FG (1995). Localization of human rhinovirus replication in the upper respiratory tract by in situ hybridization. J Infect Dis.

[B29] Bardin PG, Johnston SL, Sanderson G, Robinson BS, Pickett MA, Fraenkel DJ, Holgate ST (1994). Detection of rhinovirus infection of the nasal mucosa by oligonucleotide in situ hybridization. Am J Respir Cell Mol Biol.

[B30] Proud D, Sanders SP, Wiehler S (2004). Human rhinovirus infection induces airway epithelial cell production of human beta-defensin 2 both in vitro and in vivo. J Immunol.

[B31] Spurrell JC, Wiehler S, Zaheer RS, Sanders SP, Proud D (2005). Human airway epithelial cells produce IP-10 (CXCL10) in vitro and in vivo upon rhinovirus infection. Am J Physiol Lung Cell Mol Physiol.

[B32] Jakiela B, Brockman-Schneider R, Amineva S, Lee WM, Gern JE (2008). Basal cells of differentiated bronchial epithelium are more susceptible to rhinovirus infection. Am J Respir Cell Mol Biol.

[B33] Dreschers S, Dumitru CA, Adams C, Gulbins E (2007). The cold case: are rhinoviruses perfectly adapted pathogens?. Cell Mol Life Sci.

[B34] Reed LJ, Muench H (1938). A simple method of estimating fifty percent endpoints. Am J Hyg.

